# Spatial pattern of schistosomiasis in Xingzi, Jiangxi Province, China: the effects of environmental factors

**DOI:** 10.1186/1756-3305-6-214

**Published:** 2013-07-24

**Authors:** Yi Hu, Zhijie Zhang, Yue Chen, Zengliang Wang, Jie Gao, Bo Tao, Qiulin Jiang, Qingwu Jiang

**Affiliations:** 1 Department of Epidemiology, School of Public Health, Fudan University, Shanghai 200032, China; 2 Key Laboratory of Public Health Safety, Ministry of Education, Shanghai 200032, China; 3 Laboratory for Spatial Analysis and Modeling, School of Public Health, Fudan University, Shanghai 200032, China; 4 Biomedical Statistical Center, Fudan University, Shanghai 200032, China; 5 Department of Epidemiology and Community Medicine, Faculty of Medicine, University of Ottawa, 451 Smyth Rd, Ottawa, Ontario, Canada; 6 Xingzi Station for Schitosomiasis Prevention and Control, Xingzi, Jiangxi Province 332800, China

**Keywords:** *Schistosoma japonicum*, *Oncomelania hupensis*, Geostatistical prediction, Environmental factors, Geographic information systems

## Abstract

**Background:**

The recent rebounds of schistosomiasis in the middle and lower reaches of the Yangtze River pose a challenge to the current control strategies. In this study, identification of potential high risk snail habitats was proposed, as an alternative sustainable control strategy, in Xingzi County, China. Parasitological data from standardized surveys were available for 36,208 locals (aged between 6–65 years) from 42 sample villages across the county and used in combination with environmental data to investigate the spatial pattern of schistosomiasis risks.

**Methods:**

Environmental factors measured at village level were examined as possible risk factors by fitting a logistic regression model to schsitosomiasis risk. The approach of ordinary kriging was then used to predict the prevalence of schistosomiasis over the whole county.

**Results:**

Risk analysis indicated that distance to snail habitat and wetland, rainfall, land surface temperature, hours of daylight, and vegetation are significantly associated with infection and the residual spatial pattern of infection showed no spatial correlation. The predictive map illustrated that high risk regions were located close to Beng Lake, Liaohuachi Lake, and Shixia Lake.

**Conclusions:**

Those significant environmental factors can perfectly explain spatial variation in infection and the high risk snail habitats delineated by the predicted map of schistosomiasis risks will help local decision-makers to develop a more sustainable control strategy.

## Background

Schistosomiasis japonica, caused by *Schistosoma japonicum*, was one of the most serious parasitic diseases in China with a documented history of over 2,100 years [1]. Over the past six decades, China has made great strides toward reducing the prevalence of schistosomiasis japonica, largely through a strategy based on chemotherapy and snail control [2]. Despite this achievement, schistosomiasis has re-emerged in the past decade due to changes of biological, ecological and socio-economic factors and the termination of the World Bank Loan Project (WBLP) on schistosomiasis control in 2001 [1]. A recent review reported that approximately one million people remained infected and more than 50 million people were at risk, especially those living in the marsh and lake (Dongting Lake and Poyang Lake) regions along the Yangtze River basin [3]. The re-emergence of schistosomiasis poses a challenge to the sustainability of current implemented control strategies [4,5].

It is imperative to adopt an alternative sustainable control strategy in the lake and marshland regions in China. Chemotherapy was the backbone of previous control programs; however, repeated drug therapy resulted in a reduction in chemotherapy compliance [6]. In addition, large scale chemotherapy is no longer possible because the financial support for schistosomiasis control is generally not enough although it has increased since 2009 [7]. The transmission of *S. japonicum* is closely related to the distribution of its sole intermediate host - *Oncomelania hupensis*, which controls the distribution of schistosomiasis. However, large-scale control strategies for snail habitats over a whole endemic area are impractical [8]. The identification of areas at high risk of transmission is of great importance in the efficient control schistosomiasis [8,9].

In this study conducted in Xingzi County, we firstly mapped the spatial distribution of schistosomiasis infection, then analyzed its relevant environmental determinants, and finally identified potential high risk snail habitats.

## Methods

### Study area

Xingzi County is located in the northern part of Jiangxi Province, adjacent to the Poyang Lake, the largest freshwater lake in China. It has a total population of 25 million with 16 million living in the areas affected by schistosomiasis. Ideal snail habitats are common in Xingzi County; these are characterized by habitats described as “land in winter, water in summer”. The water level rises from April to October (wet season) to submerge the whole substratum upon which the snails live (making the area unsuitable for snail activity). During the dry season (mainly from November to the following March) water levels in the marshlands gradually subside and the grassy wet-lands favored by the snails re-emerge. This provides an ideal environment for snail growth and reproduction, which leads Xingzi County to be one of the most affected regions for schistosomisis historically.

### Data sources

#### Parasitological data

**Figure 1:**
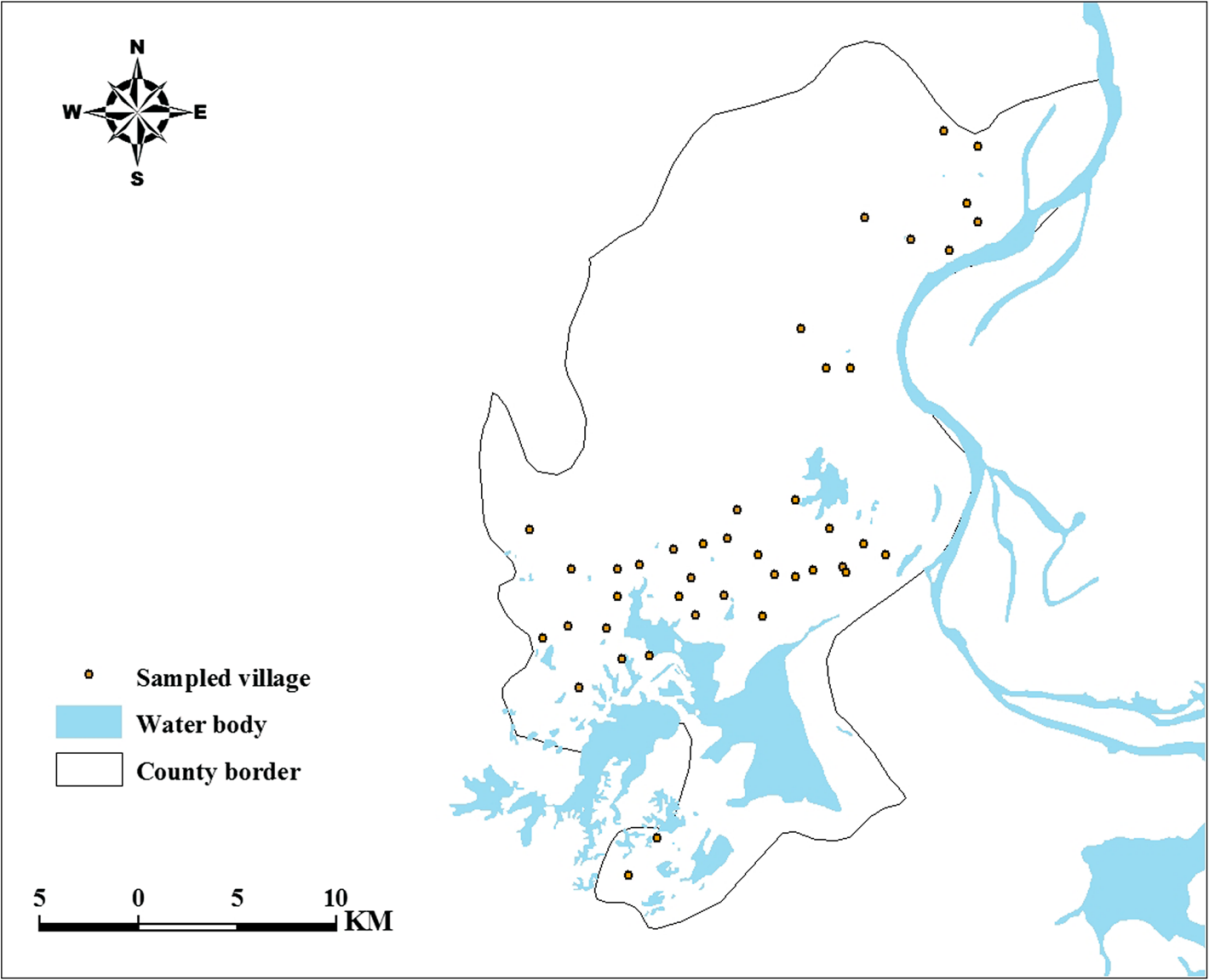
**Locations of sampled villages in Xingzi County.** Detailed legends: This map shows where the sampled villages are located in the study area.

*S. japonicum* infection data were obtained from a cross-sectional survey conducted by health professional of the local anti-schistosomiasis station at 42 randomly selected locations in November 2008. The locations of surveyed villages are shown in Figure 1. A total of 36,208 residents aged 6–65 years participated in the survey. Indirect Hemagglutination Assay (IHA) test was first used to screen the individuals according to the manufacturer’s instructions and then the parasitological test was applied to the seropositive individuals by reading three Kato-Katz thick smears from one stool specimen. All those individuals found to be egg-positive were diagnosed with S. japonicum infection. Approval for oral consent and other aspects of this survey was granted by the Ethics Committee of Fudan University (ID: IRB#2011-03-0295).

### Environmental data

#### Rainfall and hours of daylight

Monthly data of rainfall and hours of daylight in 2008 were obtained from China Meteorological Data Sharing Service System (http://cdc.cma.gov.cn/home.do). With available data at 756 meteorological stations in China, Inverse Distance Weighting (IDW) interpolation was used to derive data at locations of sampled villages. Four indexes (the minimum, the maximum, mean, and standard deviation) were calculated for each village for the consideration that together they capture, albeit crudely, the effects of overall climate condition and seasonal variation in local climate.

#### Land Surface Temperature (LST) and vegetation index

All 8-day global 1 km products for LST in 2008 and monthly global 1 km products for vegetation index (Normalized Different Vegetation Index, NDVI) that covered Xingzi County were downloaded from the Level 1 and Atmosphere Archive and Distribution System (http://ladsweb.nascom.nasa.gov/data/search.html). Similarly, the four indexes (the minimum, the maximum, mean, and standard deviation) were obtained for each village as explained above.

#### Topological data

Altitude of sampled villages were obtained from an interpolated Digital Elevation Model (DEM) from the Global Land Information System (GLIS) of the United States Geological Survey (http://egsc.usgs.gov/isb/pubs/factsheets/fs06994.html).

#### Distance to snail habitats and water body

Locations of snail habitats were recorded using handhold Global Positioning System (GPS). Data on water body, including lake and wetland, were downloaded from Conservation Science Data Sets of World Wild Foundation at http://worldwildlife.org. For each sample village, the shortest Euclidian distance to snail habitats and water body were calculated respectively.

#### Population density

Information on population density was derived from Center for International Earth Science Information Network (CIESIN) at Columbia University (http://sedac.ciesin.columbia.edu/data/collection/gpw-v3). All the data manipulation and calculation were carried out in the ArcGIS 10 (ESRI, Redlands, CA).

### Statistical analysis

Logistic regression models were fitted to schistosomasis data to identify significant environmental covariates. Initially, univariate analyses were conducted and variables with *P*>0.2 were excluded. Colinearity was investigated among all possible pairs of potential predictor variables and if any pair had a correlation coefficient >|0.2|, the member of the pair that was thought less likely to be biologically important was excluded. With the remaining variables, backwards-stepwise regression was conducted using *P* >0.1 as the exit criterion and *P* ≤0.05 as the entry criterion. Finally, the residuals from this model were tested for spatial autocorrelation using variogram [10], which will be introduced later in model-based geostatistical model. If the residuals show no spatial autocorrelation over the study area, this traditional (non-spatial) model was chosen as the fitting model; otherwise, the following model-based geostatistics model was used instead.

#### Model-based geostatistical model

Let *Y*_*i*_ denotes the number of positive samples out of *n*_*i*_ individuals tested at village *i*. The model assumes that the *Y*_*i*_ are conditionally independent binomial variates given an unobserved spatial stochastic process S(•), and that the mean response at *i* depends on explanatory variables observed at village *i* and on S(*i*). The spatial random-effect (S(i)) was modeled as a stationary Gaussian process (signal) with the mean 0, variance *σ*^*2*^ and correlation function *ρ(u)*, where *u* is the distance between two villages. The exponential correlation function used here is defined as follows:

(1) $$p\left(u\right)=\exp\left(-u/\phi \right)$$

in which *ϕ* >0 is a scale parameter with the dimensions of distance, measuring the rate at which the spatial correlation decays over distance. Under this function, the practical range (defined as the minimum distance at which spatial correlation between locations is below 5%) is approximately 3 *ϕ*. Hence if *p*(*i*) is the probability that a randomly selected person at a village *i* will test positive for schistosomiasis, then

(2) $$\log\left\{p\left(i\right)/\left[1,-,p,\left(i\right)\right]\right\}=\overset{⌒}{\mu }\left(i\right)+S\left(i\right)+\epsilon \left(i\right)$$

where *μ*(*i*) refers to a trend surface model or covariate information is available and *ϵ*(*i*) is the error term which is assumed to be mutually independent *N*(0, *τ*^2^). In geostatistics, *ϵ*(*i*) refers to a discontinuity at the origin in the variogram in geostatistical practice [10]. The role of S(i) in the model is to capture residual spatial variation after adjusting for covariates, without which equation (2) becomes traditional (non-spatial) logit model.

Finally, the predicted map of prevalence of infection with *Schistosoma japonicum* was produced and overlaid with locations of snail habitats. The ordinary kriging [11] with the Langevin-Hastings conditional simulation algorithm [12] was used to generate samples from the predicted distribution of the complete surface S(i), given the observed values of the response variable *Y*_*i*_ at each sampled village location. The Markov chain was run for 11,000 iterations to perform conditional simulation. After an initial burn-in of 1,000 iterations, the other 10,000 iterations were run and sampled every 10th iteration to generate the predicted distribution of the risk surface and the corresponding variance surface. The convergence was judged by looking at the Markov chain traces of the model parameters. The traces differed in the extent to which they showed good mixing, but they all exhibited a reasonable degree of convergence to a stationary distribution.

All the statistical analysis was implemented using R2.14.1 software (R Development Core Team, Vienna, Austria).

## Results

**Figure 2:**
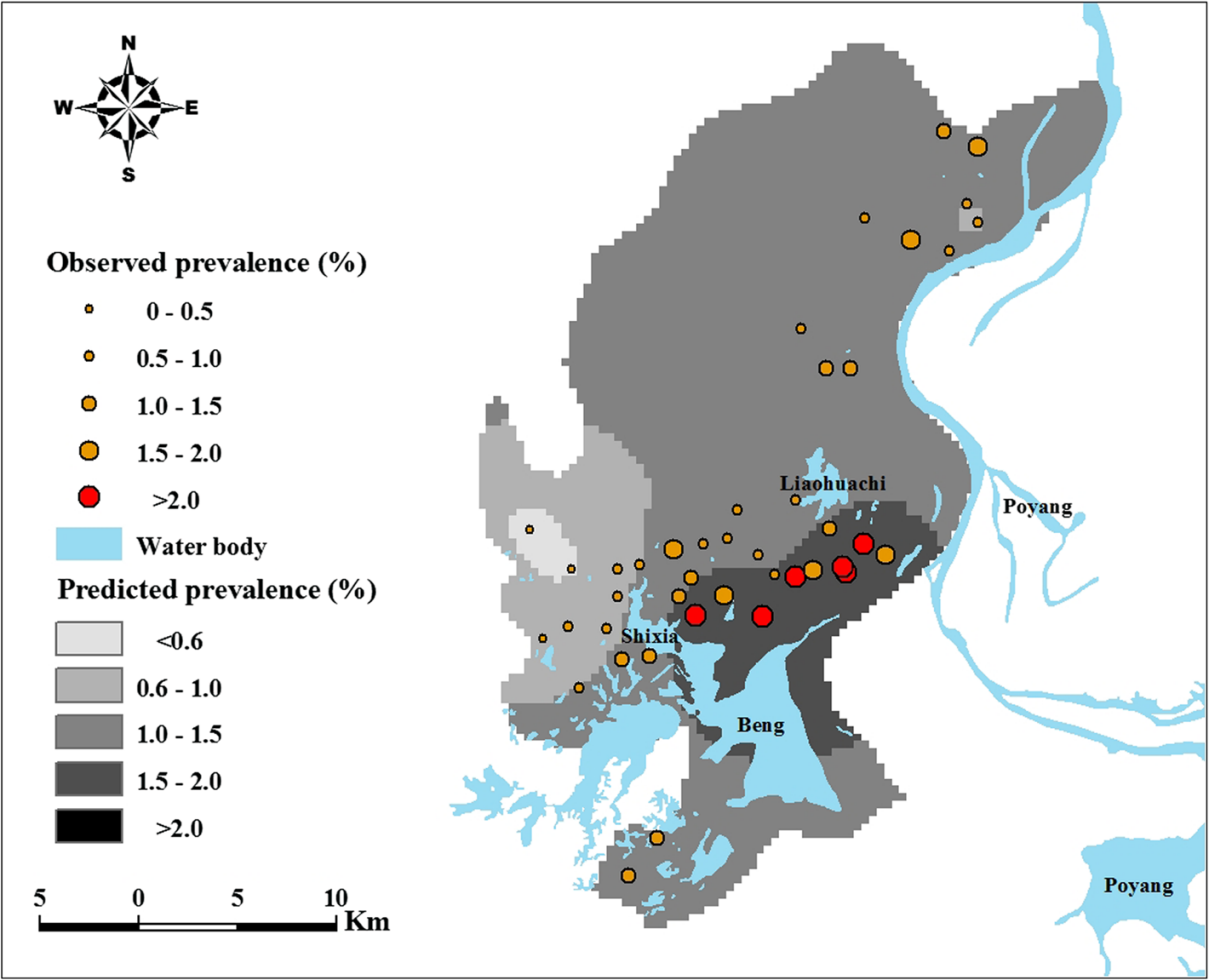
**Predicted prevalence of schistosomiasis in Xingzi County.** Detailed legends: This predictive map, over-laid with the observed prevalence in sampled villages, shows the overall prevalence of schistosomiasis over the study area. Beng, Liaohuachi, Shixia, and Poyang denote name of lakes.

Observed prevalence of S. japonicum infection for the 42 villages ranged from 0% to 9.12%, with the median of 1.08%. Figure 2 displays the spatial variation in schistosomiasis risk and shows that most of the villages were located along the shores of the major lakes, that villages closer to the lakes tended to have a higher prevalence, and that there seems a trend of reduction in risk moving from northeast to southwest.

Table 1 reports the results of fitting the data to a logistic regression model. Statistics for the covariate regression coefficients and spatial component of residuals from this model are summarized. Note that both *σ*^2^ and *ϕ* are less than 0.01, indicating that almost no spatial trend exists of the infection data after adjusting for the environmental factors. Model coefficients show that distance to snail habitats and wetland, maximum and minimum rainfall, and mean hours of sunshine were significantly negatively associated with schistosomiasis risk while the maximum LST at daytime, the maximum and minimum LST at night, and mean NDVI were significantly positively associated with schistosomiasis.

**Table 1:** Environmental determinants of S japonica: logistic regression model

**Parameters**	**Estimate**	**Standard error**	***z *value**	***p*-value**
Intercept	33.85	65.49	0.52	0.61
Distance to snail habitat	−0.11e-03	0.05e-03	−2.11	0.03
Distance to wetland	−0.45e-03	0.10e-03	−4.46	<0.01
Rainfall (min)	−7.37	1.85	−3.98	<0.01
Rainfall (max)	−0.02	9.16 e-03	−2.55	0.01
Hours of daylight (mean)	−0.57	0.15	−3.88	<0.01
LST at daytime (max)	0.37	0.01	3.79	<0.01
LST at night (min)	0.02	3.53 e-03	5.01	<0.01
LST at night (max)	0.16	0.04	3.66	<0.01
Difference of LST between daytime and night (max)	−3.06e-03	1.76 e-03	−1.74	0.08
NDVI (mean)	5.65	1.39	4.07	<0.01
*τ*^2^	0.42	---	---	---
*σ*^2^ (residuals)	<0.01	---	---	---
*ϕ* (residuals)	<0.01	---	---	---

Min: minimum; max: maxium.

**Figure 3:**
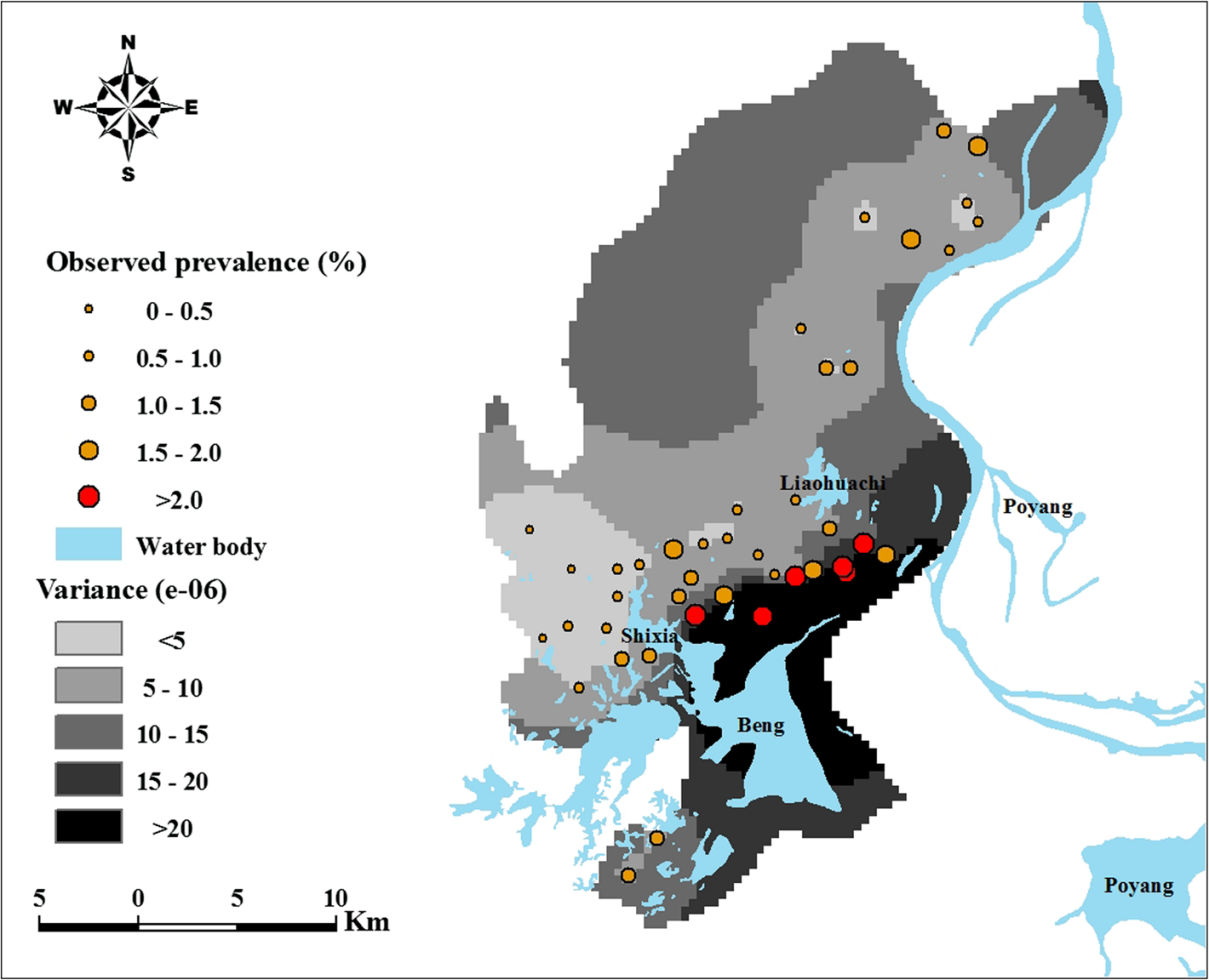
**Uncertainties of the predictions of schistosomiasis in Xingzi County.** Detailed legends: This predictive map, over-laid with the observed prevalence in sampled villages, shows uncertainties of predictions of schistosomiasis over the study area. Beng, Liaohuachi, Shixia, and Poyang denote name of lakes.

**Figure 4:**
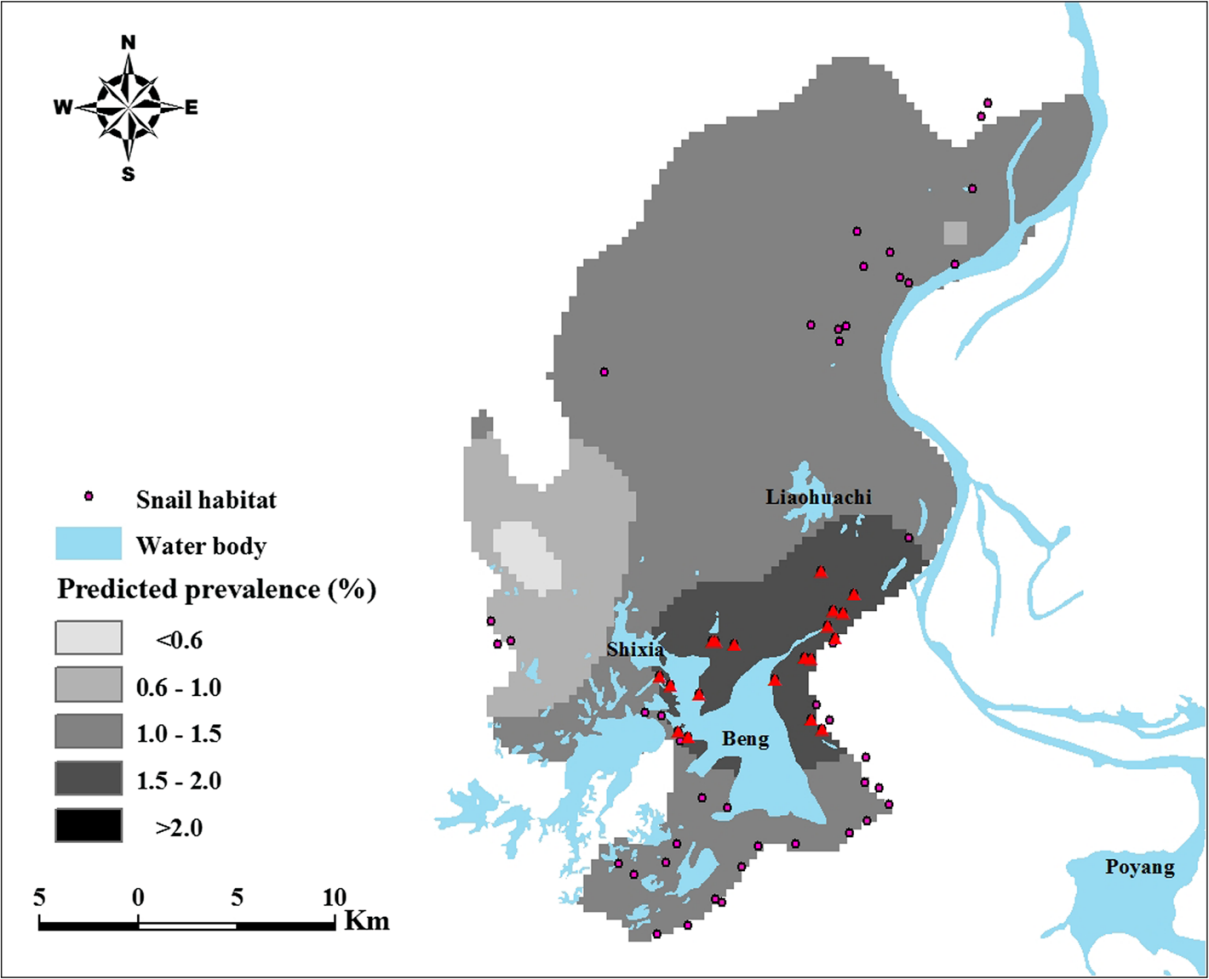
**Locations of high risk snail habitats in Xingzi County.** Detailed legends: The high risk snail habitats are marked with red triangle and Beng, Liaohuachi, Shixia, and Poyang denote name of lakes.

The map of predicted prevalence for *S. japonicum* infection (Figure 2) shows relatively high schistosomiasis prevalence (>1.5%) in the southeastern part of the county surrounded by Beng Lake, Liaohuachi Lake, and Shixia Lake. Figure 3 represents estimates of the variance of the predictions. As shown in Figure 3, a lower level of uncertainty was apparent in locations close to sampled villages and a higher level of uncertainty was apparent in locations distant from sampled villages. An exception to this was the areas around Beng Lake where the prediction uncertainty is high though some sampled villages are nearby Beng Lake. The prediction uncertainty, however, is generally low over the study area. A comparison of Figure 3 with Figure 2 shows that prediction variances were large at locations where the predictions themselves are high. The map of snail habitat overlaid by the predicted *S. japonicum* infection risk is illustrated in Figure 4. Within areas with high predicted prevalence, a portion of snail habitats (marked with triangle) were densely located between Beng Lake, Liaohuachi Lake, and Shixia Lake.

## Discussion

This study represents an application of GIS and RS techniques and geostatistics to predict *S. japonicum* infection risks in Xingzi County, one of the most serious endemic regions of schistosomiasis in China, where re-emergences and transmission of japonicum were still ongoing [1]. This study depicted the spatial pattern of *S. japonicum* infection and identified the important role of environmental factors in explaining this geographical variation. The predicted map of schistosomiasis risk provides an empirical basis for identifying prior areas when implementing schistosomiasis controls in local regions.

The observed risk factors found to be related to *S. japonicum* infection are already well known and are consistent and interpretable with the epidemiology of infection and known biology of freshwater snails. Malacological studies confirm that the habitats of *O. hupensis* snail species in lower reaches of the Yangtze River include lake/marshland regions and hill regions, both of which have extensive physical connections with the Yangtze River through channels or in low floodplains beside the Yangtze River [13,14]. With frequent floodings of the Poyang Lake due to its connection to the Yangtze River, snails in these habitats can be dispersed and subsequently deposited widely in various localities, such as rivers, lakes, and wetland. Hence, individuals living on or near the shore are more likely to undertake risky water contact behavior as a result of agricultural activities and fishing, increasing their exposure to *S. japonicum*. The distances to snail habitats and wetland could be regarded as the indicators of exposure because people tend to contact these environments more frequently if they live nearby. This finding is consistent with previous reports [8,15]. Climatic condition, such as LST, rainfall, and hours of daylight, has been shown to influence the distribution and density of snails and the rate of schistosomal development in the snail host [16-18], which is supported by our results. The NDVI is the most important vegetation index and has been widely and successfully used for prediction of intermediate host snails of schistosomiasis [19]. Higher NDVI indicates higher abundance of green vegetation which could be used as a proxy for suitable *O. hupensis* habitats, thus facilitating transmission.

Re-emergence of schistosomiasis indicates that maintaining and consolidating the achievements obtained needs a further adjustment. It is well known that the intermediate host --- snail, is an important part of the life cycle of *S. japonicum*, therefore, controlling snails is an efficient way to keep long-term sustainable control effects [20,21]. To achieve this, it is important to locate potential high risk snail habitats and take corresponding effective control strategies, such as mollusciciding and environmental modification. The predicted map of schistosomiasis risk (Figure 2) shows that regions with a relatively high prevalence of >1.5% are mainly around lakes, indicating snail habitats within these regions (marked with triangle in Figure 4) are the targets for action. There is a relatively high level of uncertainty in regions with high prevalence however. When disease maps are used in planning control programs, it is important to acknowledge such uncertainties when interpreting maps for disease control [22].

Our study highlights two limitations which deserve further discussions. One is that our prediction model relies on an assumption of stationarity. This assumption is appropriate when mapping disease over small areas, but questionable over wide areas, such as a country [23]. And geostatistical methods for modeling non-stationarity data have not well developed in spite of some studies [24,25]. However, to investigate the effects of non-stationarity is interesting and challenging and needs to be considered in further studies. The other potential limitation is that there might be diagnostic errors of infection. Generally low infection with *S. japonica* in recent years results in uncertainty in both sensitivity and specificity of infection [26]. Wang el at [27] used a Bayesian method deal with this uncertainty in a case study. The assumption of this method is that the diagnostic sensitivity and specificity for every village are all the same, which are impractical. In addition, this method might introduce new errors because of subjective prior knowledge for model parameters which heavily influences the corresponding posterior distributions [28]. Modeling with diagnostic errors is of consideration in future studies.

## Conclusion

In summary, this study investigated a region where schistosomiasis endemic remained an important public health problem. The results revealed the spatial patterns of *S. japonicum* infection in Xingzi County, which help understand its epidemiological characteristics. More importantly, we identified locations of high risk snail habitats where control efforts should be taken as a strategic priority. These results will help in the development of long-term and sustainable strategies for schistosomiasis control.

## Competing interests

All authors declare that they have no competing interests.

## Authors’ contributions

ZJ and QJ conceived the study; ZL, JG, BT, and QJ performed the field collections; YH, ZJ, and YC wrote the manuscript; YH performed statistical analyses. All the authors read and approved the final version of the manuscript.
